# A Turning Point for China’s Population: No Child and Long Illness

**DOI:** 10.14336/AD.2023.0326

**Published:** 2023-12-01

**Authors:** Wenjie Sun, Jeffery A Jones, Michelle Gamber

**Affiliations:** ^1^Department of Rural Health, Oklahoma State University Center for Health Sciences, Tulsa, OK74135, USA.; ^2^Department of Health Policy and Community Health, Jiann-Ping Hsu College of Public Health, Georgia Southern University, Statesboro, GA, USA.; ^3^School of Health Professions, Shenandoah University, Winchester, Virginia.

**To the Editor**,

According to the Chinese National Bureau of Statistics, on Jan 17, 2023, China's population fell by 850,000 people in 2022 to 1.41 billion (www.cnbc.com/2023/01/17/chinas-population-drops-for-the-first-time-in-decades.html). This is the first time the population has declined since the 1967 famine. The decline has occurred rapidly, brought about by deaths from the COVID pandemic and a drop in fertility rates that have been declining for half a century.

This shift poses health challenges for China. An old Chinese adage says: “Jiu Bing Chuang Qian Wu Xiao Zi” meaning “filial piety runs out when a parent’s sickness lasts a long time”. For Chinese families consisting of two married only children trying to both work and care for 4 elderly parents, having no siblings puts more pressure on China's only children. With Chinese life expectancy at 78.2 years in 2022, and the prevalence of disabilities among the elderly at 26.2% (95% CI: 23.7-28.6%) [1], Chinese parents have long life expectancies but require assistance. While 2.34% of Chinese aged over 60 years require assistance from a caregiver for daily behaviors [2], elderly depend heavily on their children for care due to a lack of care infrastructure such as nursing homes and geriatric services. Finally, the rapidity of the growth and sheer size of China’s graying population creates unique challenges.

“Wei Fu Xian Lao” is another Chinese idiom meaning “getting old before getting rich” [2]. This idiom underscores the health crisis facing China as millions of its people age with relatively low incomes. These millions will need extensive healthcare services from a health system with limited resources; particularly in rural areas where seniors are concentrated due to younger people moving to cities for work and opportunities. In contrast to developed countries, nursing home facilities are not widely available in China, and there is a cultural reluctance to utilize this type of care. In China, it is the children’s responsibility to take care of elderly parents. Filial piety is a centuries-old Chinese core value for the whole family [3].

Now, China’s remarkable population decline challenges expectations of filial piety and represents the long-term consequences of the 1978 One Child policy. Today, a modern Chinese family consists of two only children who are married and now face expectations to care for four elderly parents while also working and potentially caring for their own children. Urbanization has resulted in a decline in traditional family structures such as living in the same house with an extended family and the shared work of the entire extended family caring for seniors. Women used to serve as the primary caregivers of seniors, and with decades of economic expansion, women have increasingly entered the workforce as well as sought greater opportunities through education [4, 5]. This pattern of increasing opportunities for women outside of the home and the resulting decline in fertility is a pattern seen historically in other developed and industrialized countries.

To date, there has not been a successful model for handling the scenario of an aging population in a country as large as China. However, other East Asian countries have experienced similar challenges, such as South Korea, which also places a cultural emphasis on filial piety and has undergone a transition to a graying society with dropping fertility rates. Unfortunately, South Korea reports a high prevalence of suicide among the elderly as the country struggles to align an aging population, contemporary family structures, an industrialized economy, and traditional values around senior care [6]. While suicide among the elderly conflicts greatly with filial piety in Chinese culture, suicide rates in China dramatically increase with age, with the rate peaking for those over 75 years old [7].

## Solutions

Other countries offer possible solutions or lessons for China's senior care crisis. One solution is to expand the development of nursing home facilities, which contribute to successful aging in older adults. Until the 1990s, nursing facilities in China only provided services for low-income and childless seniors [8]. Private investment in nursing home facilities is needed, although it is more likely a non-profit project due to the long-term nature of such investments and low return rates. Indeed, expanding stepped senior care facilities nationwide is best handled by national Chinese health agencies. Like programs in other industrialized countries, such facilities would serve a continuum from senior daycare while family members are working to full skilled nursing facilities for seniors needing 24/7 care.

Chinese families already have the custom of hiring Hugong, trained caregivers hired by a family to sit with a family member at a hospital. In effect, these individuals serve as aides, but China lacks national standards and certification programs for geriatrics. While some Chinese universities are seeking to expand their geriatric training, such standards and policies should be a national priority [9]. There is a huge demand for these home-based services for the elderly, and after receiving systematic training on senior care, the Hugong can be an effective supplement for family care of seniors.

China is a world leader in technology, and adaptive technologies can empower seniors to assist in their own care. Voice-activated controls for lights, thermostats, and other devices can provide safe ways for seniors to remain more independent even with mobility issues. The COVID pandemic’s push to expand telehealth and remote socializing through video chat offers promises for ways for seniors to maintain and grow social and care networks without needing transportation. For caregivers, remote work has been widely accepted by many institutions and companies during the COVID pandemic, and such arrangements can be leveraged to provide more work-life balance for Chinese workers. Although it might not enough to address the complex and interrelated challenges that China's population is facing, leveraging technology and new work-life patterns can help.

On the policy level, China has loosened its restrictions on the number of children that couples can have. Moreover, Chinese couples will need slightly more than 2 children per household to maintain the status quo. It remains to be seen whether Chinese families will choose to have larger families now that such families are legal. The population decline in China could lead to a potential labor shortage for the country, which could alter the global economy by diminishing China's role as the world's factory for consumer goods.

Now, however, China must grapple with a nation of only children struggling to juggle work and traditional expectations to care for parents and other childless relatives. In many cases these children live in cities far from the rural areas where their relatively low-income parents live. Many families throughout the industrialized world can empathize with this predicament. China’s care network for seniors is especially thin and the nursing home model predominate in other industrialized countries conflicts with traditional Chinese values of filial piety. Yet, the demographic trends are clear that China must put its ingenuity to work in grappling with how a modern China will fulfill one of its most ancient values to care for elderly family members.
